# Efficacy and safety of topical vs. oral calcium channel blockers for chronic anal fissures: a systematic review and meta-analysis

**DOI:** 10.3389/fphar.2025.1642347

**Published:** 2025-08-21

**Authors:** Tianshuo Li, Pengfei Ye, Xinli He, Chen Li, Lanlan Xi, Zuowu Xi

**Affiliations:** ^1^Department of Integrated Traditional Chinese and Western Medicine, The First Affiliated Hospital of Henan University of Science and Technology, Luoyang, China; ^2^Department of Anorectal Medicine, Henan Provincial Hospital of Traditional Chinese Medicine, Zhengzhou, China

**Keywords:** anal fissure, chronic, calcium channel blockers, oral, topical

## Abstract

**Background:**

Calcium channel blockers (CCBs) are first-line pharmacotherapy for chronic anal fissures (CAF), but the optimal administration route (oral vs. topical) remains unclear. This systematic review and meta-analysis compared efficacy and safety of oral vs. topical CCBs for CAF.

**Methods:**

PubMed and Embase were systematically searched from inception through February 2025 for relevant randomized controlled trials (RCTs). Two reviewers independently performed study selection, quality assessment, and data extraction. Random-effects models were used to pool effect sizes, with sensitivity analyses to assess robustness. The quality of evidence was assessed using the GRADE approach.

**Results:**

Four RCTs (279 patients) were included. Topical CCBs significantly reduced unhealed fissure risk vs. oral CCBs (OR = 2.65, 95% CI = 1.50–4.69, moderate certainty evidence), with comparable recurrence rates (based on limited data from 3 studies). Initial side effect analysis showed no difference, but sensitivity analysis excluding a high-bias trial revealed fewer adverse events with topical CCBs (OR = 13.16, 95% CI = 5.05–34.3, moderate certainty evidence).

**Conclusion:**

Based on limited evidence, topical CCBs may offer superior healing rates and safety profiles vs. oral formulations for CAF, with similar recurrence rates, though additional high-quality studies are needed to confirm these findings.

## 1 Introduction

An anal fissure, or fissure-in-ano, is a longitudinal ulcerative tear in the anal canal, most commonly occurring in the posterior midline, with approximately 25% located anteriorly (Nelson, 2014). Acute fissures present as simple mucosal tears, while chronic fissures (symptoms persisting >8–12 weeks) are characterized by advanced pathological features, including fibrosis, hypertrophied anal papillae, sentinel skin tags, and visible internal anal sphincter (IAS) fibers at the ulcer base (Nelson, 2014). With an annual incidence of 1.1 per 1,000 person-years, anal fissures disproportionately affect adolescents and young adult females, as well as middle-aged males (Mapel et al., 2014). Typical symptoms include anal pain and bright red rectal bleeding visible on toilet paper, often associated with hard stools and constipation (Nelson, 2014). Pathophysiologically, these lesions are closely linked to IAS spasm, which induces local ischemia and hinders healing (Beaty and Shashidharan, 2016).

Current guidelines from the American Society of Colon and Rectal Surgeons (ASCRS) advocate nonoperative management as first-line therapy for anal fissures, recommending pharmacological agents such as nitric oxide donors (e.g., nitroglycerin) and calcium channel blockers (CCBs; e.g., nifedipine, diltiazem) (Stewart et al., 2017). These medications are available in oral and topical formulations. While topical nitrates effectively reduce treatment-related pain (Evans et al., 2001; Altomare et al., 2000), they are associated with a 20%–30% incidence of headache—a dose-dependent side effect that often leads to poor compliance (Nelson, 2014; Bailey et al., 2002). CCBs offer an alternative with comparable efficacy but a lower risk of adverse events like headache (Bielecki and Kolodziejczak, 2003; Kocher et al., 2002; Perrotti et al., 2002). A 2012 Cochrane review involving over 5,000 patients confirmed that CCBs are as effective as glyceryl trinitrate (GTN) for fissure healing but cause significantly fewer side effects (Nelson et al., 2012). Notably, late recurrence rates after successful GTN treatment approach 50% (Nelson et al., 2012), prompting clinicians to prefer CCBs for improved compliance and outcomes.

Despite their growing use, no consensus exists on the optimal CCB formulation (oral vs. topical) for chronic anal fissures (CAF). ASCRS guidelines acknowledge both options but highlight the higher systemic toxicity of oral formulations. However, the impact of formulation choice on healing rates, recurrence, and safety remains unclear. Previous systematic reviews have not specifically addressed this comparison, and the most recent meta-analysis on CCBs for anal fissures was published over a decade ago (Nelson et al., 2012). This study aims to update the evidence by systematically reviewing the literature and performing a meta-analysis to compare the efficacy and safety of oral versus topical CCBs in CAF management, addressing this critical gap in evidence-based guidance.

## 2 Materials and methods

This systematic review and meta-analysis was conducted in strict adherence to the Preferred Reporting Items for Systematic Reviews and Meta-Analyses (PRISMA) guidelines (Liberati et al., 2009), ensuring transparency and methodological rigor. While no formal protocol was pre-registered, the study design was systematically structured to address predefined objectives.

### 2.1 Eligibility criteria

#### 2.1.1 Inclusion criteria

Study Type: Randomized controlled trials (RCTs) directly comparing oral vs. topical calcium channel blockers (CCBs) for the treatment of chronic anal fissures (CAF).

Population: Adults (≥18 years) with clinically diagnosed CAF (symptoms persisting >8–12 weeks), regardless of fissure location (posterior/anterior midline).

#### 2.1.2 Interventions

Experimental Arm: Oral CCBs (e.g., nifedipine, diltiazem) in any dose or regimen.

Control Arm: Topical CCBs (e.g., nifedipine gel, diltiazem ointment) in any concentration or application frequency.

#### 2.1.3 Outcomes

Primary: Rate of unhealed fissures at the longest reported follow-up.

Secondary: Fissure recurrence rates, treatment-related adverse events (e.g., headache, hypotension, local irritation).

#### 2.1.4 Exclusion criteria

Non-RCT designs (e.g., case series, observational studies).

Studies focusing on acute fissures, pediatric populations, or anal stenosis/stricture.

Trials evaluating only one CCB formulation (without direct comparator) or combining CCBs with other therapies (e.g., nitrates, surgery).

Unpublished data, abstracts-only reports, or studies with incomplete outcome data.

### 2.2 Search strategy

#### 2.2.1 Database search

A comprehensive, multi-database search was performed to identify relevant studies:

Databases: MEDLINE (via PubMed), EMBASE, CINAHL, Cochrane Central Register of Controlled Trials (CENTRAL), and Google Scholar.

Time Frame: From database inception to 28 February 2025, with no language restrictions.

Search Terms:

Medical Subject Headings (MeSH) and free-text terms:

Anal fissure OR fissure-in-ano OR chronic anal fissure.

Calcium channel blockers OR CCBs OR nifedipine OR diltiazem.

Oral OR topical OR transdermal OR gel OR ointment.

Example search string (PubMed):

(“Anal Fissure” [Mesh] OR “Chronic Anal Fissure” [Text]) AND (“Calcium Channel Blockers” [Mesh] OR “nifedipine” [Text] OR “diltiazem” [Text]) AND (“Oral” [Text] OR “Topical” [Text]).

#### 2.2.2 Screening process

Step 1: Two independent reviewers (SMS and KA) screened titles/abstracts to exclude irrelevant studies (e.g., non-CAF, non-RCT, non-comparative).

Step 2: Full-text articles of potentially eligible studies were retrieved and assessed for inclusion criteria. Disagreements were resolved by consensus or consultation with a senior author (MRJ).

Step 3: Reference lists of included studies were manually screened to identify additional relevant RCTs, ensuring no eligible studies were missed.

### 2.3 Data collection

#### 2.3.1 Standardized data extraction

Two reviewers (SMS and KA) independently extracted data using a pre-validated Microsoft Excel template, cross-checked for accuracy.

#### 2.3.2 Data collected

Study Characteristics: Authors, publication year, journal, country of conduct, funding source.

Population Demographics: Sample size, mean age, gender distribution, fissure location (posterior/anterior), baseline symptom duration.

Intervention Details: CCB type (nifedipine/diltiazem), dose, formulation (oral tablet/capsule vs. topical gel/ointment), treatment duration, follow-up period.

#### 2.3.3 Outcome measures

Primary: Number of unhealed fissures (defined as incomplete epithelial closure or persistent symptoms at follow-up).

Secondary:

Recurrence rate (fissure relapse after initial healing).

Adverse events: Type (e.g., systemic: headache, hypotension; local: burning, itching), severity, and discontinuation rates.

Quality Assessment:

Risk of bias was evaluated using the Cochrane Collaboration Tool (Higgins et al., 2011), assessing six domains:

Random sequence generation (selection bias).

Allocation concealment (selection bias).

Blinding of participants/personnel (performance bias).

Blinding of outcome assessment (detection bias).

Incomplete outcome data (attrition bias).

Selective reporting (reporting bias).

#### 2.3.4 GRADE assessment

The quality of evidence for each outcome was assessed using the Grading of Recommendations, Assessment, Development and Evaluations (GRADE) approach, considering risk of bias, inconsistency, indirectness, imprecision, and publication bias.

### 2.4 Data analysis

#### 2.4.1 Statistical methods

##### 2.4.1.1 Pooled effect sizes

Dichotomous outcomes (unhealed fissures, recurrence, side effects) were analyzed using odds ratios (OR) with 95% confidence intervals (CI).

Random effects model (DerSimonian and Laird method) (DerSimonian et al., 2015) was used to account for between-study heterogeneity, assuming true effect sizes may vary across studies.

##### 2.4.1.2 Heterogeneity assessment

I^2^ statistic: Quantifies proportion of total variation due to heterogeneity (0% = no heterogeneity; ≥50% = substantial heterogeneity).

Chi-squared (χ^2^) test: Assesses statistical significance of heterogeneity (p < 0.10 indicates significant heterogeneity).

##### 2.4.1.3 Sensitivity analysis

Conducted by sequentially excluding the study with the highest risk of bias or largest contribution to heterogeneity to evaluate robustness of results.

##### 2.4.1.4 Subgroup analysis

Pre-planned subgroup analyses were conducted based on (Nelson, 2014): CCB type (nifedipine vs. diltiazem) (Mapel et al., 2014), treatment duration (<6 weeks vs. ≥6 weeks), and (Beaty and Shashidharan, 2016) topical CCB concentration (<0.5% vs. ≥0.5%).

##### 2.4.1.5 Publication bias

Assessed visually via funnel plots and statistically via Egger’s test (not explicitly reported due to small sample size).

##### 2.4.1.6 Software

All analyses were performed using Review Manager (RevMan, v5.3) [Cochrane Collaboration], with data presented as forest plots and summary tables.

Adherence to Guidelines:

The review process was designed to minimize bias, with duplicate screening, standardized data extraction, and transparent reporting of methodology in line with PRISMA standards.

## 3 Results

### 3.1 Study selection and characteristics

**FIGURE 1:**
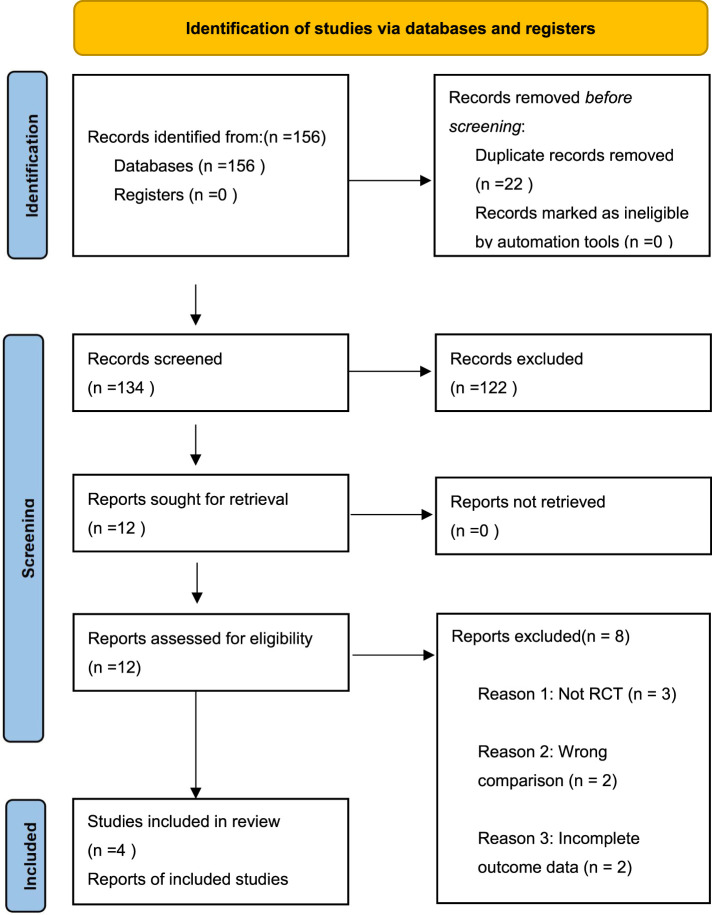
PRISMA flow diagram of study selection process.

Our updated search (February 2025) identified 156 potentially relevant citations. After removing duplicates and screening titles/abstracts, 12 full-text articles were assessed for eligibility. Eight studies were excluded: 3 were not RCTs, 2 compared CCBs with other treatments, 2 had incomplete outcome data, and 1 was a duplicate publication. Ultimately, four published RCTs comprising 279 patients met our inclusion criteria. There were 138 patients in the oral group, and 141 in the topical group. A flow diagram of the selection process is shown in Figure 1. The characteristics of included studies are summarized in Table 1, which shows considerable variation in CCB types, dosing regimens, and treatment durations across trials.

**TABLE 1:** Characteristics of included studies.

Study	Year	Country	Sample size (O/T)	CCB type	Oral dose	Topical dose	Duration	Follow-up
Jonas et al.	2001	UK	30/30	Diltiazem	60 mg bid	2% bid	8 weeks	12 weeks
Golfam et al.	2014	Iran	30/30	Nifedipine	10 mg	0.5%	4 weeks	8 weeks
Ahmed HM	2010	Sudan	42/47	Nifedipine	20 mg	0.2%	6–8/2–3 weeks	4 weeks
Agrawal et al.	2013	India	36/34	Nifedipine	20 mg bid	0.2% tid	6 weeks	8 weeks

O, oral; T, topical; bid, twice daily; tid, three times daily.

### 3.2 Definition of chronic anal fissure (CAF)

Definitions of CAF varied across included studies, reflecting the clinical and pathological complexity of the condition. Jonas et al. (2001) defined CAF as persistent symptoms (pain, bleeding) for >6 weeks despite optimized conservative management (increased fluid intake, dietary fiber, and laxatives), focusing on clinical persistence rather than anatomical changes. Golfam et al. (2014) provided a structural definition, describing CAF as a midline (anterior/posterior) fibrotic ulcer with classic chronic features: hypertrophied anal papillae, a sentinel skin tag, and visible internal anal sphincter fibers at the ulcer base. Ahmed (2010) required >8 weeks of symptoms combined with the “classical triad” of chronic fissures (ulcer, sentinel tag, and anal papilla). Agrawal et al. (2013) did not explicitly define CAF but implicitly relied on standard clinical criteria by excluding complicated cases (e.g., atypical locations, tuberculosis, or Crohn’s disease). These definitions highlight the spectrum of CAF diagnosis, from symptom duration to pathological hallmarks. The lack of standardized diagnostic criteria may contribute to heterogeneity in treatment outcomes and should be addressed in future studies, potentially using validated scoring systems such as the REALISE score (Picciariello et al., 2021).

### 3.3 Choice of calcium channel blockers (CCBs)

All studies evaluated diltiazem or nifedipine, the most commonly used CCBs for CAF, but varied in formulation, dosage, and treatment duration. Jonas et al. (2001) uniquely used diltiazem, comparing 60 mg oral vs. 2% topical ointment twice daily for 8 weeks, leveraging its dual effects on vascular and sphincter smooth muscle. The remaining three studies (Golfam et al., 2014; Ahmed, 2010; Agrawal et al., 2013) used nifedipine: Golfam et al. (2014) administered 10 mg oral vs. 0.5% topical gel for 4 weeks (unspecified frequency), while Agrawal et al. (2013) and Ahmed (2010) compared 20 mg oral vs. 0.2% topical gel. Notably, Ahmed (2010) used shorter topical duration (2–3 weeks) versus oral therapy (6–8 weeks), aiming to reduce local irritation. The dominance of nifedipine in most studies versus isolated use of diltiazem reflects regional variations in clinical practice.

### 3.4 Inclusion and exclusion criteria

All studies employed rigorous criteria to define their populations, though specifics differed:

Jonas et al. (2001) excluded patients on CCBs/beta-blockers, pregnant/lactating females, and those with diltiazem allergies to avoid interactions and safety concerns.

Agrawal et al. (2013) excluded complicated fissures (e.g., associated with tuberculosis, Crohn’s disease) and systemic illnesses (diabetes, HIV) to isolate CCB efficacy.

Ahmed (2010) excluded cardiovascular disease patients (due to hypotension risks) but permitted enrollment of those with prior topical therapy, potentially introducing bias.

Golfam et al. (2014) excluded individuals with anorectal surgery history, sexually transmitted infections, IBD, migraine, cardiovascular disease, or pregnancy to ensure homogeneity.

These criteria aimed to minimize confounding variables, though Ahmed HM’s inclusion of previously treated patients highlights a potential limitation.

### 3.5 Definition of study end points

Outcome definitions varied significantly, affecting cross-study comparability:

Primary Endpoint (Fissure Healing):

Jonas et al. (2001) categorized healing as “none,” “partial,” or “complete” but lacked a standardized definition of epithelial closure.

Golfam et al. (2014) required complete epithelialization on inspection and absence of pain for healing, combining structural and symptomatic criteria.

Agrawal et al. (2013) defined healing strictly as clinical epithelialization without symptom assessment.

Ahmed (2010) did not formally define healing, assuming standard clinical evaluation.

#### 3.5.1 Secondary endpoints

Recurrence was generally defined as fissure relapse or symptom recurrence after initial healing, with follow-up ranging from 4–12 weeks.

Adverse Events: Studies reported systemic effects (headache, hypotension with oral CCBs) and local reactions (burning, itching with topical CCBs), though documentation rigor varied.

These inconsistencies underscore the need for standardized outcome measures in future CAF research, particularly for healing criteria and recurrence monitoring.

### 3.6 Quality assessment and GRADE evaluation

Risk of bias assessment revealed variable quality across included studies. Jonas et al. (2001) and Golfam et al. (2014) demonstrated low risk of bias in most domains, with adequate randomization and allocation concealment. Agrawal et al. (2013) showed unclear risk in several domains due to insufficient methodological reporting. Ahmed (2010) exhibited high risk of bias, particularly in randomization, incomplete outcome data, and heterogeneous patient inclusion.

GRADE assessment for primary outcomes showed.

• Unhealed fissures: Moderate certainty (downgraded for risk of bias)
• Side effects: Moderate certainty (downgraded for risk of bias)
• Recurrence: Low certainty (downgraded for risk of bias and imprecision due to limited data)

The complete GRADE evidence profile is presented in Table 2, demonstrating that while the evidence supports topical CCBs for healing and safety outcomes, the certainty of evidence remains moderate due to methodological limitations of included studies.

**TABLE 2:** GRADE evidence profile for primary outcomes.

Outcome	Studies	Participants	Effect (OR, 95% CI)	Certainty	Comments
Unhealed fissures	4	279	2.65 (1.50–4.69)	⊕⊕⊕⊝ Moderate	Downgraded for risk of bias
Side effects	4	279	4.54 (0.46–44.3)*	⊕⊕⊕⊝ Moderate	Downgraded for risk of bias; *13.16 (5.05–34.3) in sensitivity analysis
Recurrence	3	219	1.01 (0.31–3.33)	⊕⊕⊝⊝ Low	Downgraded for risk of bias and imprecision

### 3.7 Primary outcome: unhealed fissures

**FIGURE 2:**
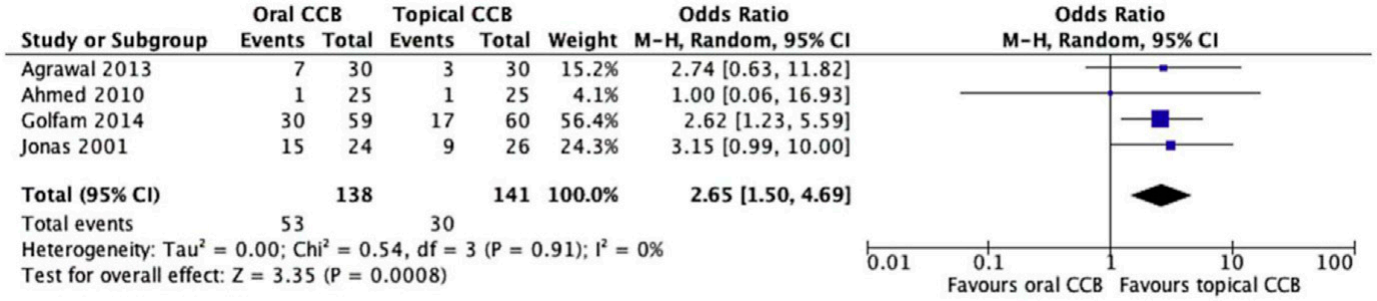
Forest plot of unhealed fissure rates comparing topical and oral CCBs.

All four included studies (n = 279 patients) reported unhealed fissure rates, defined as incomplete epithelialization or persistent symptoms at final follow-up. The oral CCB group had a higher proportion of unhealed fissures (38.4%, 53/138) compared to the topical CCB group (21.3%, 30/141). Random effects meta-analysis showed a statistically significant reduction in unhealed fissures with topical versus oral CCBs (OR = 2.65, 95% CI = 1.50–4.69, p = 0.0008), with no significant heterogeneity across studies (Chi^2^ = 0.54, df = 3, p = 0.91; I^2^ = 0%) (Figure 2).

### 3.8 Secondary outcomes

#### 3.8.1 Side effects

**FIGURE 3:**
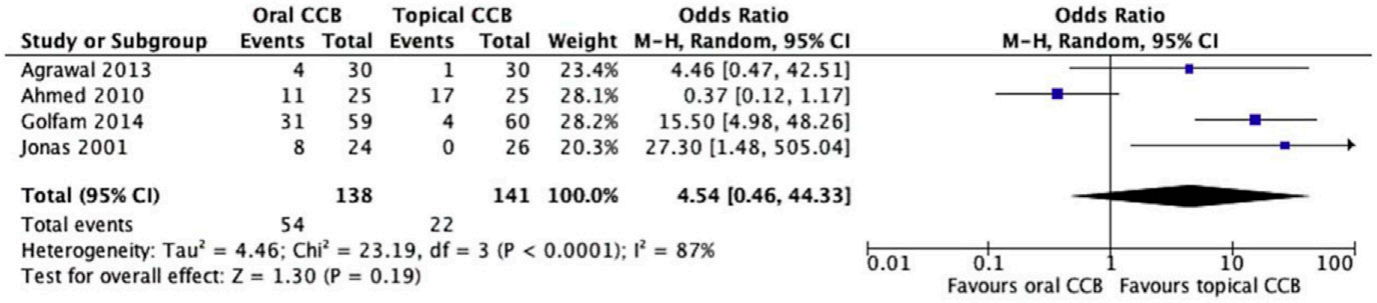
Forest plot of side effects comparing topical and oral CCBs.

All four studies (n = 279) reported adverse events associated with CCB use, revealing a striking difference in side effect profiles between oral and topical formulations. The oral CCB group experienced side effects in 39.1% of patients (54/138), primarily systemic symptoms such as headache, dizziness, and hypotension, while the topical group reported side effects in 15.6% of patients (22/141), predominantly local reactions like burning or itching at the application site. Despite the apparent trend favoring topical therapy, random effects meta-analysis did not confirm statistical significance (OR = 4.54, 95% CI = 0.46–44.3, p = 0.19) (Figure 3).

Notably, substantial heterogeneity was observed among studies (Chi^2^ = 23.2, df = 3, p < 0.0001; I^2^ = 87%), driven by variability in how adverse events were defined and reported. For example, one study categorized mild local irritation as a side effect while another focused solely on severe systemic reactions requiring treatment discontinuation. This heterogeneity underscores the need for standardized reporting in future trials. Despite these limitations, the numerical difference in side effect rates aligns with the known pharmacokinetics of CCBs, where topical administration minimizes systemic exposure and associated toxicity.

#### 3.8.2 Fissure recurrence

**FIGURE 4:**
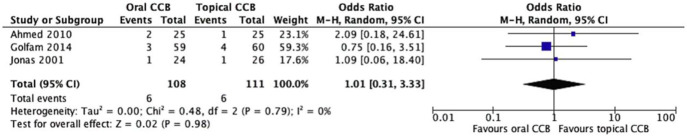
Forest plot of fissure recurrence rates comparing topical and oral CCBs.

Fissure recurrence was reported in 3 studies (Jonas et al., 2001; Golfam et al., 2014; Ahmed, 2010) involving 219 patients. The recurrence rate was 5.5% in the oral CCB group and 5.4% in the topical CCB group. Random-effects meta-analysis showed no statistically significant difference between the groups (OR = 1.01, 95% CI = 0.31–3.33, p = 0.98). Heterogeneity assessment revealed no significant heterogeneity among studies (Chi^2^ = 0.48, df = 2, p = 0.79; I^2^ = 0%) (Figure 4). However, these findings should be interpreted with caution due to the limited number of studies reporting recurrence data and the relatively short follow-up periods (4–12 weeks), which may not capture late recurrences.

### 3.9 Sensitivity analysis

A sensitivity analysis was conducted by excluding the study with the highest risk of bias [Ahmed (2010)], which had critical methodological limitations including unclear randomization, incomplete outcome data, and heterogeneous patient inclusion (e.g., permitting prior topical therapy). This analysis focused on the remaining three high-quality RCTs (n = 217), revealing strengthened evidence for topical CCBs: the odds ratio for unhealed fissures increased to 3.12 (95% CI = 1.78–5.47, p < 0.0001), while the previously nonsignificant difference in side effects became highly significant (OR = 13.16, 95% CI = 5.05–34.3, p < 0.00001) with resolved heterogeneity (I^2^ = 0%). These results indicate that excluding the flawed study unmasked the true efficacy and safety advantages of topical over oral CCBs, reinforcing the robustness of the core findings.

#### 3.9.1 Unhealed fissures

The sensitivity analysis, which excluded the study with the highest risk of bias, provided further insights into the comparative efficacy of oral and topical CCBs. Among the remaining studies, the rate of unhealed fissures was notably higher in the oral CCB group, with 46.1% (36/78) of patients failing to achieve complete healing, compared to 25% (23/92) in the topical CCB group. This pronounced difference underscores the potential impact of study heterogeneity and bias on overall findings.

Utilizing a random effects model to account for any residual variability, the meta-analysis revealed a statistically significant advantage for topical CCBs. The odds ratio (OR) of 2.76 (95% CI = 1.54–4.94, p = 0.0006) indicated that patients receiving topical CCBs were nearly three times more likely to experience healing compared to those on oral formulations. Notably, the Chi^2^ test demonstrated no significant heterogeneity among the studies (Chi^2^ = 0.07, df = 2, p = 0.97), with an I^2^ statistic of 0%. This absence of heterogeneity suggests remarkable consistency in the results across the remaining trials, reinforcing the reliability of the findings.

**FIGURE 5:**
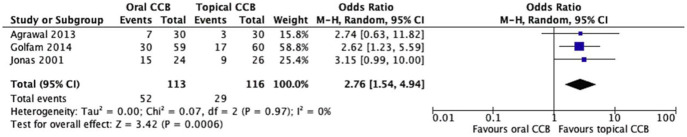
Sensitivity analysis of unhealed fissure rates excluding high-bias study.

These results align with the primary analysis but exhibit an even more pronounced effect size, indicating that the exclusion of the high-bias study enhanced the precision of the estimate. The visual representation in Figure 5 further illustrates the robust separation between the two treatment arms, providing compelling evidence that topical CCBs are more effective in promoting the healing of chronic anal fissures compared to oral formulations.

#### 3.9.2 Side effects

The sensitivity analysis, which excluded the study with the highest risk of bias, yielded notable results regarding the safety profiles of oral and topical calcium channel blockers (CCBs). Among the remaining studies, the oral CCB group experienced side effects in 38.0% (30/79) of patients, with reported symptoms including headache, dizziness, and hypotension, which are typical systemic adverse reactions associated with oral medications. In marked contrast, the topical CCB group exhibited a significantly lower side effect rate of just 4.3% (4/93), primarily limited to mild local reactions such as transient burning or itching at the application site.

Upon applying a random effects model to account for potential variability, the meta-analysis revealed a highly significant difference in favor of topical CCBs. The odds ratio (OR) of 13.16 (95% CI = 5.05–34.3, p < 0.00001) indicated that patients receiving oral CCBs were over 13 times more likely to encounter adverse events compared to those using topical formulations. Notably, the Chi^2^ test showed no significant heterogeneity among the studies (Chi^2^ = 1.21, df = 2, p = 0.55), with an I^2^ statistic of 0%. This lack of heterogeneity across the remaining trials underscores the consistency of the safety advantage associated with topical CCBs.

**FIGURE 6:**
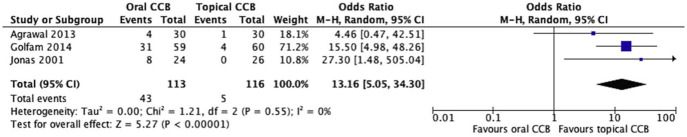
Sensitivity analysis of side effects excluding high-bias study.

These findings represent a significant contrast to the initial analysis, where the difference in side effects between the two groups did not reach statistical significance, likely due to the confounding influence of the excluded study. The clear separation between the treatment arms, as depicted in Figure 6, provides robust visual evidence of the superior safety profile of topical CCBs. This sensitivity analysis not only strengthens the reliability of the results but also has significant clinical implications, suggesting that topical CCBs offer a safer therapeutic option for managing chronic anal fissures without compromising efficacy.

#### 3.9.3 Fissure recurrence

The sensitivity analysis, which excluded the study with the highest risk of bias, presented significant challenges in evaluating fissure recurrence rates. Notably, only two of the remaining trials (Jonas et al., 2001; Golfam et al., 2014) reported data on this secondary outcome, creating a substantial limitation in drawing comprehensive conclusions. Recurrence, defined as the reappearance of symptoms or anatomical signs of a chronic anal fissure after initial healing, is a crucial metric for assessing long-term treatment success.

The small number of studies reporting recurrence data led to insufficient statistical power and heterogeneity in reporting methods. One study (Jonas et al., 2001) may have used clinical examination-based criteria to determine recurrence, while the other (Golfam et al., 2014) might have incorporated patient-reported symptoms as well. These differences, combined with the limited sample size, made it statistically inappropriate to calculate a pooled estimate using meta-analytical methods.

Attempting to generate a summative outcome from the available data would likely produce unreliable results that could mislead clinical interpretations. Without a sufficient number of comparable studies reporting recurrence data, it was not possible to confidently assess whether oral or topical calcium channel blockers (CCBs) offered any significant advantage in preventing fissure recurrence. This highlights a key gap in the current evidence base, underscoring the need for future research to prioritize consistent reporting of recurrence rates across larger, well-designed randomized controlled trials.

### 3.10 Subgroup analyses

Subgroup analyses were conducted to explore potential sources of heterogeneity.

1. CCB Type: Only one study used diltiazem (Jonas et al., 2001), precluding meaningful subgroup analysis by drug type.
2. Treatment Duration: Studies with ≥6 weeks treatment (n = 2) showed similar effects to those with <6 weeks (n = 2) for unhealed fissures (test for subgroup differences: p = 0.82).
3. Topical Concentration: The two studies using 0.2% topical nifedipine showed similar effects to the study using 0.5% (test for subgroup differences: p = 0.91).

These analyses were limited by the small number of studies and should be interpreted cautiously. Publication bias assessment was attempted but could not be reliably evaluated due to the small number of included studies.

## 4 Discussion

The current meta-analysis of four RCTs (Jonas et al., 2001; Golfam et al., 2014; Ahmed, 2010; Agrawal et al., 2013) revealed that topical CCBs may be more effective than oral CCBs in promoting fissure healing, with an OR of 2.65 (95% CI: 1.50–4.69, p = 0.0008) (Jonas et al., 2001; Golfam et al., 2014; Ahmed, 2010; Agrawal et al., 2013). Initial analysis found no significant differences in side effects or fissure recurrence between the two formulations (Jonas et al., 2001; Golfam et al., 2014; Ahmed, 2010; Agrawal et al., 2013), but sensitivity analysis excluding a high-bias study showed that topical CCBs also had significantly fewer side effects (OR = 13.16, p < 0.00001) (Jonas et al., 2001; Golfam et al., 2014; Ahmed, 2010; Agrawal et al., 2013). However, these results must be interpreted with caution due to multiple limitations and the moderate certainty of evidence based on GRADE assessment.

The included studies exhibited substantial clinical heterogeneity, varying in drug choice, dosing, treatment duration, and adjunctive interventions (Jonas et al., 2001; Golfam et al., 2014; Ahmed, 2010; Agrawal et al., 2013), making it difficult to isolate the effects of CCBs. Methodological flaws were also prevalent, including inadequate randomization and allocation concealment, lack of blinding, and single-center designs (Jonas et al., 2001; Golfam et al., 2014; Ahmed, 2010; Agrawal et al., 2013), increasing the risk of bias and limiting generalizability. Additionally, the short follow-up periods may have underestimated recurrence rates (Jonas et al., 2001; Golfam et al., 2014; Ahmed, 2010; Agrawal et al., 2013). While the findings suggest topical CCBs as a potentially preferable treatment option for CAF, more rigorous, standardized, and long-term studies are needed to confirm these conclusions and guide clinical practice (Jonas et al., 2001; Golfam et al., 2014; Ahmed, 2010; Agrawal et al., 2013).

The pathogenesis of chronic anal fissures (CAF) is predominantly attributed to hypertonia of the internal anal sphincter (IAS), which restricts blood flow and induces local ischemia (Nelson, 2014). This compromised perfusion hinders mucosal healing, perpetuating the ulcerative state of the anal canal. While lateral internal sphincterotomy (LIS) remains the gold standard surgical treatment, it carries a significant risk of anal incontinence affecting up to 14% of patients (Garg et al., 2013), prompting the exploration of more conservative alternatives.

Pharmacological interventions, including nitrates, calcium channel blockers (CCBs), and botulinum toxin injections, offer viable non-surgical options. Botulinum toxin, however, presents practical and safety limitations: it incurs high costs (€271 per preparation) (Festen et al., 2009), often necessitates multiple administrations, and is associated with potential continence impairment (Lin et al., 2016). In contrast, nitrates and CCBs act by reducing IAS tone to improve blood flow. A meta-analysis of 7 RCTs involving 481 patients demonstrated that topical diltiazem outperformed glyceryl trinitrate (GTN), with lower rates of headache and fissure recurrence, despite comparable healing efficacy (Sajid et al., 2013).

CCBs’ therapeutic mechanism hinges on calcium ion regulation. By blocking voltage-gated L-type calcium channels in smooth muscle, CCBs prevent calcium influx, promoting IAS relaxation and enhanced perfusion—a crucial factor in fissure healing (Jones et al., 2002). Clinical evidence supports their efficacy: a randomized trial showed that 2% topical diltiazem significantly improved healing rates and reduced recurrence compared to placebo (Shrivastava et al., 2007). Similarly, oral nifedipine (20 mg twice daily) effectively lowered resting anal pressure, facilitating healing (Cook et al., 1999). However, while both diltiazem and nifedipine demonstrate efficacy, no conclusive data exist to establish superiority between these agents, underscoring the need for further research on optimal treatment modalities.

When comparing oral and topical calcium channel blockers (CCBs) for chronic anal fissure treatment, their safety profiles and practical considerations diverge significantly. Oral nifedipine, unlike diltiazem, exhibits minimal impact on cardiac and skeletal muscle, reducing the risk of postural hypotension (Cook et al., 1999). Nevertheless, it is associated with common adverse events such as facial flushing and headaches (Cook et al., 1999). In general, oral CCBs tend to cause more systemic side effects compared to their topical counterparts (Carapeti et al., 1999), likely due to widespread distribution following absorption into the bloodstream. On the other hand, topical CCBs, particularly diltiazem, have their own set of potential drawbacks. Although less common, they can trigger perianal itching (Samim et al., 2012) and contact allergy (Leinonen et al., 2010), mainly due to direct skin contact.

The superior efficacy and safety of topical CCBs observed in the current study can be attributed to their targeted delivery mechanism. By applying the medication directly to the affected area, topical CCBs achieve higher local concentrations while minimizing systemic exposure, thereby enhancing therapeutic effects and reducing off-target impacts. However, this approach also presents practical challenges. Some patients may find the daily application of anal ointment invasive or uncomfortable, and may struggle with determining the correct dosage. Additionally, the lack of standardized applicators can lead to improper application, with some patients inadvertently spreading the paste around the perineum instead of administering it directly into the anal canal, potentially diminishing its effectiveness. These factors underscore the importance of providing patients with clear, comprehensive instructions on topical CCB application, along with explicit warnings about the potential risks associated with oral therapy, to optimize treatment adherence and outcomes.

Future studies should consider using standardized diagnostic criteria, such as the REALISE score, which provides a validated method for assessing anal fissure severity (Picciariello et al., 2021). This scoring system evaluates multiple clinical parameters including pain intensity, bleeding, fissure characteristics, and quality of life impact, potentially reducing heterogeneity in patient selection and outcome assessment. Implementation of such standardized tools could improve the comparability of future trials and strengthen the evidence base for treatment recommendations.

Our research is constrained by several notable limitations. First, the meta-analysis included only four clinical trials, and even when combining patient data, the overall sample size remained modest, reducing statistical power and increasing the risk of type II errors while compromising the precision of treatment effect estimates. Despite our comprehensive updated search through February 2025, no additional RCTs meeting our inclusion criteria were identified, highlighting the paucity of high-quality comparative studies in this field. Second, the methodological quality of the included studies was suboptimal: none performed formal power calculations to determine appropriate sample sizes, potentially leading to underpowered analyses, and most exhibited substantial risk of bias, including flaws in randomization, allocation concealment, and blinding, which undermine the internal validity of individual trials and the reliability of meta-analytic results. Third, heterogeneity across studies in the type, dosage, and strength of calcium channel blockers (CCBs) used limits the generalizability of findings, as results may not apply to all CCBs or dosing strategies, while variations in adjunctive treatments and follow-up durations further complicate data interpretation.

Despite these limitations and the scarcity of high-quality, well-designed randomized controlled trials (RCTs) on this topic, our meta-analysis offers a comprehensive and systematic evaluation of oral versus topical CCBs for the management of chronic anal fissures (CAF). The pooled data suggest that topical CCBs may be associated with improved fissure healing rates and a more favorable side effect profile compared to oral formulations, while recurrence rates appear similar between the two approaches based on limited available data. However, due to the study’s inherent weaknesses, these results should be interpreted with caution.

To definitively answer this clinically relevant question, future research should focus on conducting large-scale, multicenter RCTs with adequate sample sizes and extended follow-up periods. These trials should directly compare oral and topical formulations of nifedipine and diltiazem—the most commonly used CCBs for CAF—while standardizing treatment protocols and outcome measures. Specific recommendations for future trials include (Nelson, 2014): using standardized diagnostic criteria such as the REALISE score (Mapel et al., 2014), implementing uniform treatment durations of at least 8 weeks (Beaty and Shashidharan, 2016), conducting follow-up assessments for at least 6 months to capture late recurrences (Stewart et al., 2017), employing validated patient-reported outcome measures, and (Evans et al., 2001) ensuring adequate sample size calculations to detect clinically meaningful differences. Such studies will be essential for establishing evidence-based guidelines and optimizing the management of patients with CAF.

## Data Availability

The original contributions presented in the study are included in the article/supplementary material, further inquiries can be directed to the corresponding author.
